# Salivary diagnostic markers in males and females during rest and exercise

**DOI:** 10.1186/s12970-017-0185-8

**Published:** 2017-08-10

**Authors:** Kay Rutherfurd-Markwick, Carlene Starck, Deborah K. Dulson, Ajmol Ali

**Affiliations:** 1 School of Health Sciences, Massey University, Auckland, New Zealand; 2 Centre for Metabolic Health Research, Massey University, Palmerston North, New Zealand; 3 Riddet Institute Massey University, Palmerston North, New Zealand; 4 Sports Performance Research Institute New Zealand, Auckland University of Technology, Auckland, New Zealand; 5 School of Sport, Exercise and Nutrition Massey University, Auckland, New Zealand

**Keywords:** Hydration, Stress response, Immune markers, Electrolytes, Sex

## Abstract

**Background:**

Saliva is a useful diagnostic tool for analysis in sports, exercise and nutrition research, as collection is easy and non-invasive and it contains a large number of analytes affected by a range of physiological and pathological stressors and conditions. This study examined key salivary electrolytes and stress and immune markers in males and females at rest and during exercise.

**Methods:**

Unstimulated whole saliva from 20 healthy, recreationally active participants (8 males and 12 females) was analysed for flow rate, osmolality, sodium (Na^+^), potassium (K^+^), chloride (Cl^−^), secretory immunoglobulin A (SIgA), α-amylase activity and cortisol during both rest and moderate intensity (70% peak power) cycling exercise in a randomised crossover design. Each trial lasted 60 min and sampling was carried out at 15 and 45 min after the start of the trial. Saliva was collected using the gold-standard drool method; participants were required to provide at least 1 mL sample over 2 or 3-min period.

**Results:**

Females showed a greater response to steady-state exercise stress than males, with significant increases in osmolality (*P* < 0.001), α-amylase activity (*P* = 0.001) and secretion rate (*P* = 0.023) and SIgA secretion rate (*P* = 0.023), with trends for an increase in K^+^ (*P* = 0.053) and decrease in Cl− (*P* = 0.067). There were no differences between rest and exercise for any salivary analytes in males. In addition, females showed a trend for higher levels of cortisol than males at both rest (*P* = 0.099) and exercise (*P* = 0.070), as well as a higher heart rate (*P* < 0.001) and greater ratings of perceived exertion (*P* < 0.001) during the exercise trial. The coordination of the two stress response pathways (α-amylase vs cortisol) was positive in males (*r* = 0.799; *P* = 0.017) yet negative in females (*r* = −0.475; *P* = 0.036).

**Conclusions:**

Males and females show a markedly different response to steady-state exercise stress as measured in unstimulated whole saliva.

## Background

Saliva is gaining momentum as a relevant fluid for clinical and forensic diagnosis, as well as for analysis in sports, exercise and nutrition research, as collection is easy and non-invasive and it contains a large number of analytes affected by a range of physiological and pathological stressors and conditions [1–5]. In addition, saliva is less complex than serum, including lower protein content, thus requiring substantially less preparation for analysis [2, 3]. Furthermore, saliva may be used to examine the role of sex hormones in stress and disease [6–9].

Hydration, electrolyte status, stress and immune responses are key markers for exercise performance and health status [2, 3, 10]. A 2–3% dehydration-associated body mass loss has been linked with a reduction in heat regulation, cardiovascular function and exercise performance [11] and results in significant changes in salivary composition [1]. Water is the predominant fluid constituent of saliva, thus hypo-hydration is expected to decrease salivary flow-rate, increase osmolality, and may alter the concentrations of key electrolytes, hormones and proteins [11–13].

Exercise modulates both the innate and acquired arms of the immune system [7] and activates the two major neuroendocrine stress response arms, the hypothalamic-pituitary-adrenal (HPA) axis and the sympathetic-adreno-medullary (SAM) axis (sympathetic nervous system). While SAM activation is an immediate response to exercise, the HPA axis shows a delayed response [6, 14]. Saliva carries two primary markers of HPA and SAM activation, cortisol and α-amylase, respectively [15]. Alpha-amylase is a reliable indicator of the established blood markers for SAM, epinephrine and norepinephrine, as well as playing a role in mucosal immunity [16]. However, salivary secretory immunoglobulin A (SIgA) is the most widely recognised marker of mucosal immunity [17, 18] and, there appears to be a relationship between decreases in SIgA and increased risk of upper respiratory tract infection (URTI) [10, 19]. Immune and stress responses work together to combat exercise stress [6], with both the HPA and SAM axes modulating the function of the immune system.

While blood sampling has historically been used to measure hydration, electrolyte status and markers of stress and immunity, blood sampling procedures may not be practical for the setting, they can be expensive and the invasive approach may not be appealing for all participants [4]. The analysis of other bodily fluids such as saliva holds promise in these situations; however, our understanding of the actions and interactions of the key salivary diagnostic markers in response to stress is incomplete. Most studies have presented information about a selection of markers only and differences in methodology between studies have led to equivocal information [4]. Information in the literature is limited mostly to reviews, in which correlations have been made between studies using different types of participants and varying protocols [2, 3, 10]. Moreover, while it appears that there are sex-related differences in the response of salivary markers to exercise stress [16, 20] most of the research focuses on men or a mixed cohort [3, 10]; hence data pertaining to women in isolation, or comparing the male and female response, is scarce [3, 15].

Our aim was to conduct a thorough analysis of a wide range of salivary analytes in males and females both at rest and in response to exercise, in order to provide a valuable reference dataset for future studies.

## Methods

### Participants

In total, 20 recreationally active participants completed the study (males *n* = 8; females *n* = 12: mean age 27.4 ± 5.9 years). Males (height 1.77 ± 0.04 m; weight 81.1 ± 6.5 kg) were significantly taller and heavier than females (height 1.66 ± 0.06 m; weight 62.8 ± 8.4 kg; *P* < 0.001). All procedures had prior approval by the local institutional ethics committee. Following completion of a health screening questionnaire, written informed consent was obtained from all participants. In order to be considered for inclusion in this study participants were required to be free of injury, chronic disease and infection in the 4 weeks prior to the study.

### Preliminary procedures

A preliminary session was undertaken to familiarise participants with the experimental protocol. Upon arrival to the laboratory, participants were shown the correct technique for saliva specimen collection by the passive drool method for the collection of unstimulated whole saliva (UWS). Each participant then performed an incremental exercise test on a cycle ergometer (Ergomedic 874E, Monark Exercise AB, Vansbro Sweden) starting at 60 W, with intensity increasing by 30 W·min^−1^ until volitional fatigue. Following a brief rest of 5–10 min, participants cycled at a resistance corresponding to 70% of their previously determined peak power for 10 min, then reported their perceived exertion and level of confidence regarding completing 60 min of continuous exercise at this intensity.

### Main trials

In a randomised cross-over design, participants performed either an exercising or resting protocol; the alternative protocol was performed on their subsequent visit (3–7 days later). The exercising protocol involved 60 min of steady-state cycling at 70% peak power, whereas for the resting trial participants sat quietly for 60 min.

Participants were asked to refrain from consuming caffeine and alcohol and avoid exercise in the 24-h period prior to the trial. They were also asked to replicate the same food and beverage intake prior to each trial and report to the laboratory 3 h post-prandial. Four hours prior to their arrival to the laboratory, participants were reminded (via text message) to consume the 7 mL·kg^−1^ BM quantity of water provided by the researcher in the preliminary session. Upon arrival to the laboratory, a midstream urine sample was obtained for immediate determination of hydration status by urine specific gravity (USG) using a handheld refractometer (Sur-Ne, Atago Co Ltd., Japan); all participants’ USG levels were below 1.020 and therefore were well hydrated prior to exercise. Body mass was measured before and immediately after each trial period.

Saliva was collected via the UWS drool method at two time points (15 min and 45 min) during each protocol. Both trials were conducted at the same time of day (15:00–18:00 h) to overcome any circadian influences. Heart rate (HR; T31 Polar heart rate monitor, Kempele, Finland) was measured continuously and ratings of perceived exertion (RPE) were monitored at 10-min intervals during exercise.

### Saliva collection and analysis

Saliva was collected into a disposable pre-weighed 60 mL plastic container. Participants were instructed to sit leaning forwards with their head tilted downwards and swallow before any sampling took place. During sampling participants were asked to perform minimal orofacial movement and to allow the saliva to dribble into the tube. At least 1 mL was collected over a 2-min period per participant. If insufficient sample was obtained after this time, a further minute of collection was performed. Saliva was weighed (Sartorius LE3235, Germany) and flow rate was calculated on the assumption that saliva density was 1 g·mL^−1^ [21]. Saliva specimens were then stored at −80 °C until analysis.

Saliva osmolality was measured using a freezing point depression osmometer according to the manufacturer’s instructions (Osmomat 030, Gonotec, Berlin, Germany). Salivary electrolyte levels were measured using an EasyLyte analyser according to the manufacturer’s instructions (Medica Corporation, Bedford, MA, USA). Salivary secretory IgA concentration was determined by ELISA as described elsewhere [22]. Salivary cortisol concentration was determined by radioimmunoassay according to the manufacturer’s instructions (IBL International GMBH, Tecan, Hamburg, Germany, IBMG1206). Salivary α-amylase activity was determined using the Infinity Amylase Liquid stable reagent (Thermoscientific, Worthing, UK) according to the manufacturer’s instructions.

The secretion rates of SIgA (μg·min^−1^) and α-amylase (U·min^−1^) were calculated by multiplying the saliva flow rate (mL·min^−1^) by the IgA concentration (mg·L^−1^) and α-amylase activity (U·mL^−1^), respectively.

### Statistical analysis

Independent t-tests were used to compare data between males and females. Paired t-tests were used to compare 15 min vs. 45 min at rest and 15 min vs. 45 min during exercise. Paired t-tests were used to compare rest vs. exercise data for males and females, separately (mean of rest vs mean of exercise). Pearson’s correlation was used to examine the relationships between independent variables. The results are presented as mean values ± standard deviation. Statistical significance was accepted at *P* < 0.05.

## Results

### Exercise trial

Although there were no differences in USG between rest and exercise trials in males (*P* = 0.178) or females (*P* = 0.972), pre-exercise, females exhibited lower USG than males (*P* = 0.018). HR was higher during exercise than rest (*P* < 0.001), increased during exercise for both sexes (*P* < 0.001), but remained constant at rest. During exercise, females had a higher average HR than males (*P* < 0.001) but there were no sex differences for HR at rest (*P* = 0.136). RPE increased with duration of exercise (*P* = 0.006) and was higher at 60 min compared to 10 min (*P* < 0.05). Females reported higher average RPE than males during exercise (*P* < 0.001). There was no difference in body mass loss between males and females during exercise although females lost more fluid than males in both absolute mass (*P* = 0.014) and as a percentage of body mass (*P* = 0.001).

### Hydration parameters

**Fig. 1:**
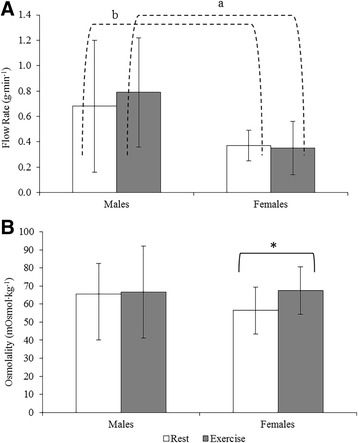
Mean data for (**a**). Flow rate (g·min^−1^) and (**b**). osmolality (mOsmol·kg^−1^) and associated errors between rest and exercise for males and females. A significant difference between rest and exercise is indicated with an asterisk (**, *P* < 0.001; *, *P* < 0.05) and between males and females with letters (*a*, *P* < 0.001; *b*, *P* < 0.05)

There was no change in UWS flow rate between rest and exercise for males (*P* = 0.248) or females (*P* = 0.801; Fig. 1a). However, males produced a higher flow rate than females during exercise (*P* = 0.007) and there was a trend for increase in flow rate at rest (*P* = 0.056). Within the exercise trial itself, females showed an increase in flow rate from 15 to 45 min (*P* = 0.031; Table 1); this was not observed in males (*P* = 0.730). However, females (*P* = 0.010) also produced a significant increase in flow rate within the rest trial (Table 1).

**Table 1:** Levels of salivary analytes at two time points during rest and exercise trials in males and females, degree of change between the time points by percentage (Δ) and the significance of this change (P)

	Rest					Exercise				
	T_1_	T_2_	Δ (%)	P		T_1_	T_2_	Δ (%)	P	
Flow rate (g · min^-1^)										
Males	0.64 ± 0.47	0.72 ± 0.57	8.9 ± 26.6	0.176		0.81 ± 0.38	0.77 ± 0.52	-1.7 ± 39.5	0.730	
Females	0.34 0.13	0.40 ± 0.12	21.3 ± 31.0	0.010	↑	0.32 ± 0.19	0.38 ± 0.24	16.7 ± 24.4	0.031	↑
Osmolality (mOsmol · kg^-1^)										
Males	65.4 ± 15.8	65.9 ± 18.7	0.31 ± 9.1	0.850		62.1 ± 18.5	71.3 ± 34.8	11.9 ± 27.1	0.287	
Females	57.3 ± 15.1	56.0 ± 11.8	-0.20 ± 15.1	0.650		66.3 ± 16.6	68.8 ± 11.2	6.1 ± 14.0	0.434	
Na (mmol · L^-1^)										
Males	4.5 ± 2.2	5.1 ± 1.8	28.3 ± 48.9	0.451		5.1 ± 2.5	9.3 ± 12.0	37.7 ± 69.8	0.285	
Females	6.4 ± 3.2	5.0 ± 1.3	-11.0 ± 31.0	0.136		5.3 ± 2.4	6.6 ± 1.8	35.9 ± 61.6	0.199	
K (mmol · L^-1^)										
Males	19.7 ± 4.1	21.3 ± 6.2	10.6 ± 40.5	0.528		22.3 ± 4.6	22.8 ± 5.1	2.2 ± 11.0	0.626	
Females	21.9 ± 6.0	19.5 ± 4.8	-9.3 ± 15.1	0.060	↓	23.5 ± 5.5	24.3 ± 5.5	3.9 ± 7.5	0.150	
Cl (mmol · L^-1^)										
Males	32.6 ± 13.2	35.9 ± 20.4	13.8 ± 64.7	0.634		32.2 ± 13.5	36.6 ± 22.6	10.6 ± 26.2	0.343	
Females	49.6 ± 27.2	43.1 ± 23.3	-9.7 ± 26.7	0.193		37.6 ± 19.1	38.7 ± 16.6	5.9 ± 14.3	0.543	
SIgA concentration (mg · L^-1^)										
Males	65.7 ± 42.2	66.8 ± 52.0	-3.5 ± 31.1	0.895		56.1 ± 41.2	61.2 ± 49.2	12.0 ± 53.4	0.526	
Females	86.4 ± 49.0	60.2 ± 35.8	8.3 ± 130.1	0.120		95.2 ± 103.9	112.9 ± 91.3	46.1 ± 63.4	0.083	↑
SIgA secretion rate (μg · min^-1^)										
Males	33.6 ± 17.5	39.1 ± 37.2	12.8 ± 59.9	0.559		39.6 ± 35.1	31.1 ± 12.9	7.5 ± 62.4	0.486	
Females	25.2 ± 17.1	24.1 ± 18.8	34.6 ± 137.1	0.275		29.7 ± 27.8	45.5 ± 34.4	78.8 ± 110.3	0.032	↑
α-amylase activity (U∙mL^-1^)										
Males	34.6 ± 20.4	38.2 ± 22.1	12.3 ± 12.2	0.050	↑	34.8 ± 19.5	44.0 ± 24.6	26.5 ± 19.0	0.034	↑
Females	25.7 ± 23.5	29.5 ± 24.9	24.9 ± 27.4	0.030	↑	41.7 ± 21.9	56.3 ± 22.3	35.2 ± 32.9	<0.001	↑
α-amylase secretion rate (U · min^-1^)										
Males	20.8 ± 18.9	27.6 ± 26.1	26.6 ± 34.4	0.559		28.1 ± 22.2	30.5 ± 22.6	22.9 ± 53.5	0.672	
Females	7.3 ± 5.9	11.3 ± 9.6	68.4 ± 51.5	0.275		13.1 ± 9.5	20.6 ± 12.1	68.4 ± 51.5	0.004	↑
Cortisol (nmol · L^-1^)										
Males	4.21 ± 0.73	4.02 ± 0.48	-3.7 ± 8.2	0.210		4.63 ± 0.71	4.55 ± 0.60	-1.0 ± 8.5	0.617	
Females	5.95 ± 2.26	5.26 ± 2.16	-11.4 ± 8.3	<0.001	↓	6.00 ± 1.88	5.96 ± 2.08	-0.3 ± 10.8	0.894	

UWS was sampled at 15 min (T_1_) and 45 min (T_2_) during each protocol. Arrows indicate whether there was an increase (↑) or decrease (↓) in the levels of the corresponding analyte over time

There was a rise in saliva osmolality for females (*P* = 0.01) during exercise compared to rest, but no change for males (*P* = 0.838) and no difference between sexes at rest (*P* = 0.191) or during exercise (*P* = 0.926; Fig. 1b). There was no difference in saliva osmolality over time within the rest or exercise trials for either sex (*P* > 0.05; Table 1).

### Electrolytes

**Fig. 2:**
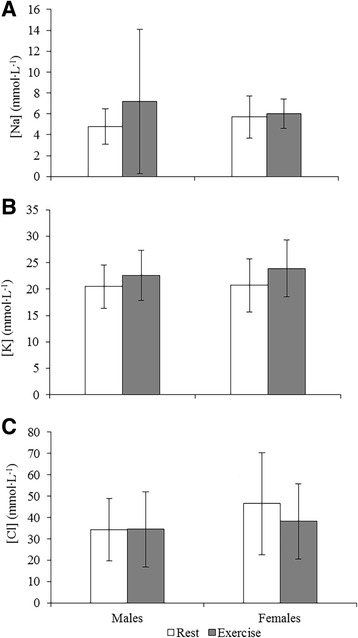
Mean electrolyte data and associated errors between rest and exercise for males and females. **a**. Na (mmol·L^−1^); **b**. K (mmol·L^−1^) and **c**. Cl (mmol·L^−1^)

There was no change in salivary Na^+^ levels between rest and exercise in males or females; nor was there any change in Na^+^ levels between males and females in the rest or exercise trials (*P* > 0.05; Fig. 2a). There was no change in saliva Na^+^ levels during the rest or exercise trials for males or females (*P* > 0.05; Table 1). Concentrations of salivary K^+^ showed a trend for an increase between rest and exercise in females (*P* = 0.053); however, there was no change in males (*P* = 0.107; Fig. 2b). While there was no change in salivary K^+^ levels within the rest or exercise trials for either sex (*P* > 0.05), there was a trend for a decrease in salivary K^+^ during rest in females (*P* = 0.060; Table 1). Salivary Cl^−^ levels showed a trend for a decrease in females between rest and exercise (*P* = 0.067); however, there was no change for males (*P* = 0.971; Fig. 2c). There was no change in salivary Cl^−^ levels during the rest or exercise trials for either sex (*P* > 0.05; Table 1).

### Mucosal immune and stress markers

**Fig. 3:**
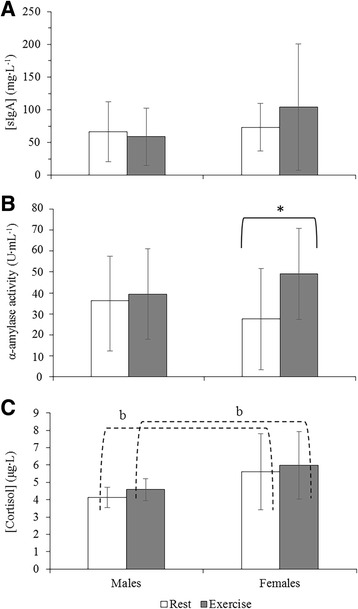
Mean data for salivary markers of (**a**). mucosal immunity, SIgA (mg·L^−1^); (**b**) the sympathetic stress response, α-amylase activity (U·mL^−1^) and **c**. the adrenal stress response, cortisol (μg·L^−1^), and associated errors between rest and exercise for males and females. A significant difference between rest and exercise is indicated with an asterisk (**, *P* < 0.001; *, *P* < 0.05) and between males and females with letters (b, *P* < 0.001; b, *P* < 0.05)

There was no change in SIgA concentration in response to exercise in males or females (*P* > 0.05); nor was there a change between the sexes at rest (*P* = 0.728) or exercise (*P* = 0.235; Fig. 3a). There was no change in SIgA concentration during the rest trial for either sex (*P* > 0.05; Table 1). There was a trend for an increase in SIgA concentration during exercise in females (*P* = 0.083). Alpha-amylase activity increased in the exercise trial compared to rest for females (*P* = 0.001; Fig. 3b) but not males (*P* = 0.501). There was no change in salivary α-amylase activity between males and females at rest (*P* = 0.429) or exercise (*P* = 0.345). However, there were significant increases in salivary α-amylase activity during both the rest and exercise trials for both sexes (*P* < 0.05; Table 1).

When expressed as a secretion rate (which takes flow rate into account) SIgA and α-amylase secretion rates increased from 15 min to 45 min during exercise in females (*P* < 0.05 for both) with α-amylase secretion rates increasing in the exercise trial compared to rest (*P* = 0.023). There were no differences between sexes at rest (*P* > 0.05) or during exercise (*P* > 0.05) for SIgA or α-amylase secretion rate.

There was a trend for higher salivary cortisol levels in females compared to males at both rest (*P* = 0.099) and during exercise (*P* = 0.070). There was no difference in salivary cortisol levels between rest and exercise for either sex (*P* > 0.05). Within the rest trial, a significant decrease in salivary cortisol was observed for females (*P* = 0.003; Table 1) but not males (*P* = 0.206). Salivary cortisol levels remained unchanged during the exercise trial.

## Discussion

The aim of this study was to examine salivary analytes in males and females both at rest and in response to exercise. The main finding was that males and females show a markedly different response to steady-state exercise stress as measured in unstimulated whole saliva. This study provides separate novel datasets of salivary responses to exercise stress in males and females that can be used as a reference point for future research.

A recognised limitation of the gold standard drool collection method [23] for UWS is the low flow rate relative to stimulated methods, and females have a lower UWS flow rate than males due to smaller salivary glands [24]. Our data showed females had lower flow rates during both exercise (*P* = 0.007) and rest (*P* = 0.056) compared to males. This difference in salivary flow rate may become limiting for data analysis, as flow rate has been suggested to influence the concentrations of some salivary analytes [1, 10, 24]. Flow rate has been suggested to be affected by exercise [10], however no difference in salivary flow rate was observed between rest and exercise for either males or females in this study.

Exercise increased salivary osmolality in females (Fig 1b); in conjunction with an increase in both α-amylase activity (Fig 3b) and secretion rate (Table 1), this supports the consistently reported exercise-driven activation of the SAM axis [16, 25]. These results were not seen in males, indicative of sex-specific differences in the salivary response to exercise stress. The reason for the increase in α-amylase activity, which was observed during exercise and rest for both sexes, is unclear but it is possible that in the absence of exercise stimulation, the antimicrobial and/or digestive roles of α-amylase may affect exercise-independent measurements. In addition, anticipation of sampling, causing premature activation of the autonomic stress response, may also affect α-amylase activity levels [6].

Unstimulated resting salivary electrolyte levels are typically in the range of 3–29 mmol·L^−1^ (Na^+^), 6.4–36.6 mmol·L^−1^ (K^+^) and 0–27 mmol·L^−1^ (Cl^−^) [2]. The broad ranges reflect the various factors which affect salivary electrolyte levels such as hydration status and salivary flow rate; the Na^+^ and K^+^ values from this study fall within normal ranges while the Cl^−^ levels are higher than the quoted ranges. Although salivary K^+^ levels increased in females in response to exercise, there were no accompanying increases in Na^+^ or Cl^−^. In fact, there was a trend for a decrease in Cl^−^ in females. While SAM activation may induce electrolyte release, research indicates substantial variation in the effects of exercise on salivary electrolyte levels which are impacted by exercise intensity and saliva collection methods [10].

In this study females showed an increase in SIgA during the exercise trial, whereas other studies have reported a decrease in SIgA in response to exercise [10]. However, SIgA may be affected by training status and the type of training carried out [10]; although we recruited recreationally active participants the inter-individual variation in SIgA measurements was large and therefore this result must be interpreted with caution.

**Fig. 4:**
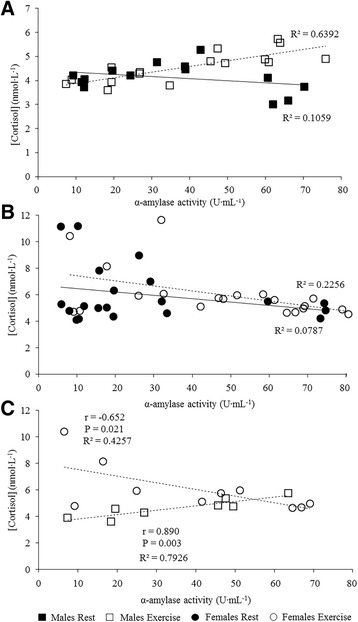
Correlations between α-amylase activity and cortisol, representing the sympathetic and adrenal stress responses, respectively, for (**a**). males; (**b**). females for both rest (filled markers) and exercise (empty markers). **c** shows a direct comparison of the exercise response at 15 min between males (*squares*) and females (*circles*)

Taken together our results suggest a notable difference in the physiological response to exercise stress between males and females, particularly with respect to activation of the SAM and HPA axes, represented by α-amylase and cortisol, respectively. While the coordination of the SAM and HPA pathways indicates a functional stress response, the activation of one without the other may represent a dysfunctional response due to the physiological consequences of chronic exposure to fluctuating or heightened neuroendocrine responses resulting from repeated or chronic stress (‘allostatic load’) [26]. Interestingly, the relationship between α-amylase activity and cortisol levels produced during exercise gave opposing trends for males and females (Fig. 4). While both sexes showed a weak but negative α-amylase versus cortisol relationship at rest, during exercise this relationship became strongly positive for males (*r* = 0.799; *P* = 0.017; Fig. 4a) yet remained negative for females (*r* = −0.475; *P* = 0.036; Fig. 4b). Considering the dysfunctional stress response to allostatic load it is unlikely that all females exhibit dysfunctional stress systems. One explanation is that the difference between males and females is complicated by the delayed response of cortisol to stress, in comparison to that of α-amylase [14]. To address this, we performed correlational analysis at 15 min and 45 min as well as a lagged comparison with α-amylase at 15 min and cortisol at 45 min. The strongest correlation for both males (*r* = 0.890; *P* = 0.003) and females (*r* = −0.652; *P* = 0.021) was observed at 15 min (Fig. 4c), indicating a result consistent with the overall exercise stress relationship. Males showed a coordinated increase in both cortisol and α-amylase, whereas females exhibited a negative relationship, indicating that the stress axes function independently.

While exercise is considered to stimulate both HPA and SAM stress response systems, the response of each is dependent on a number of factors including exercise intensity, duration, training status and the sex hormones [6]. The spread of the female α-amylase versus cortisol plot in Fig. 4c indicates three different scenarios: points in the middle of the plot indicate the two systems working together at a moderate level; points in the lower right region show sympathetic activity but no HPA response and points in the top left quartile represent HPA activity without SAM activation. Since exercise intensity and duration was consistent between the sexes, and training status is unlikely to be sex-dependent, the most likely explanation for the contrasting neuroendocrine responses observed in females involves menstrual status or menstrual cycle phase, although the latter has been repeatedly shown not to influence these pathways [27, 28]. We did not collect hormonal data, and so a detailed study into the effects of the female hormones on exercise-associated stress pathway coordination is warranted.

The stress pathways also modulate immune responses [6] with increases in the circulating stress hormones catecholamines and cortisol mediating SIgA responses to exercise. Whilst there is scarce human research it has been suggested that cortisol inhibits transepithelial transport of SIgA, while adrenaline appears to enhance IgA transcytosis [29, 30]. In the current study α-amylase was positively associated with SIgA in both males (*r* = 0.457; *P* = 0.255) and females (*r* = 0.214; *P* = 0.505), however, in males the cortisol-SIgA relationship was positive (*r* = 0.255; *P* = 0.592) while in females it was negative (*r* = −0.177; *P* = 0.440). Such weak non-significant associations limit the conclusions that can be drawn, but do lend some support to both the SAM activity and HPA axis potentially regulating SIgA response.

This study has some limitations. It is apparent from this study that there are sex-specific responses to exercise, likely due to the steroid hormones and thus, to fully understand these differences, the measurement of these hormone levels is necessary. In addition, although the drool method is considered to be the gold standard approach for diagnosis it has inherent issues, particularly a low flow rate that differs between males and females. Although menstrual cycle phase has been shown not to influence neuroendocrine responses [27, 28] future studies may wish to control for menstruation when using female participants. Our data show several differences between males and females but we also report a few non-significant trends, which may indicate that a larger sample size may have been required, and so universal conclusions cannot be made.

## Conclusions

Monitoring hydration status, exploring immune responses to exercise and examining exercise stress are important considerations for sports and exercise nutrition scientists and practitioners. Saliva sampling is becoming increasingly important with regards non-invasive monitoring of athletes as well as non-athletes. Our data provides an overview of the electrolyte, immune and stress response to steady-state submaximal exercise in both males and females using the current gold standard drool method to collect unstimulated whole saliva. This data has revealed some important differences in the response of males and females to steady-state exercise stress, particularly, opposing associations between the two major neuroendocrine stress axes. While we are unable to make specific conclusions about the mechanisms involved, future studies directly comparing exercise stress in males and females is warranted.

## Abbreviations

Cl: Chloride
HPA: Hypothalamic-pituitary-adrenal
HR: Heart rate
K^+^: Potassium
Na^+^: Sodium
RPE: Ratings of perceived exertion
SAM: Sympathetic-adreno-medullary
SIgA: Secretory immunoglobulin A
URTI: Upper respiratory tract infection
USG: Urine specific gravity
UWS: Unstimulated whole saliva

## References

[1] Kaufman E, Lamster IB (2002). The diagnostic applications of saliva- a review. Crit Rev Oral Biol Med.

[2] Nunes LAS, Mussavira S, Bindhu OS (2015). Clinical and diagnostic utility of saliva as a non-invasive diagnostic fluid:a systematic review. Biochem Med (Zagreb).

[3] Papacosta E, Nassis GP (2011). Saliva as a tool for monitoring steroid, peptide and immune markers in sport and exercise science. J Sci Med Sport.

[4] Yoshizawa JM, Schafer CA, Schafer JJ, Farrell JJ, Paster BJ, Wong DTW (2013). Salivary biomarkers: toward future clinical and diagnostic utilities. Clin Microbiol Rev.

[5] Lindsay A, Costello JT (2017). Realising the potential of urine and saliva as diagnostic tools in sport and exercise medicine. Sports Med.

[6] Fragala M, Kraemer W, Denegar C, Maresh C, Mastro A, Volek J (2011). Neuroendocrine-immune interactions and responses to exercise. Sports Med.

[7] Gillum T, Kuennen M, Schneider S, Moseley P (2011). A review of sex differences in immune function after aerobic exercise. Exerc Immunol Rev.

[8] Mastorakos G, Pavlatou M, Diamanti-Kandarakis E, Chrousos GP (2005). Exercise and the stress system. Hormones (Athens).

[9] Tiidus P (2000). Estrogen and gender effects on muscle damage, inflammation, and oxidative stress. Can J Appl Physiol.

[10] Chicharro J, Lucía A, Pérez M, Vaquero A, Ureña R (1998). Saliva composition and exercise. Sports Med.

[11] Walsh N, Laing S, Oliver S, Montague J, Walters R, Bilzon J (2004). Saliva parameters as potential indices of hydration status during acute dehydration. Med Sci Sports Exerc.

[12] Ely B, Cheuvront S, Kenefick R, Spitz MG, Heavens KR, Walsh NP, Sawka MN (2014). Assessment of extracellular dehydration using saliva osmolality. Eur J Appl Physiol.

[13] Munoz CX, Johnson EC, DeMartini JK, Huggins RA, McKenzie AL, Casa DJ, Maresh CM, Armstrong LE (2013). Assessment of hydration biomarkers including salivary osmolality during passive and active dehydration. Eur J Clin Nutr.

[14] de Vries WR, Bernards NT, de Rooij MH, Koppeschaar HP (2000). Dynamic exercise discloses different time-related responses in stress hormones. Psychosom Med.

[15] Maruyama Y, Kawano A, Okamoto S, Ando T, Ishitobi Y, Tanaka Y, Inoue A, Imanaga J, Kanehisa M, Higuma H, Ninomiya T, Tsuru J, Hanada H, Akiyoshi J (2012). Differences in salivary alpha-amylase and Cortisol responsiveness following exposure to electrical stimulation versus the Trier social stress tests. PLoS One.

[16] Rohleder N, Nater UM (2009). Determinants of salivary α-amylase in humans and methodological considerations. Psychoneuroendocrinol.

[17] Brandtzaeg P (1992). Humoral immune response patterns of human Mucosae: induction and relation to bacterial respiratory tract infections. J Infect Dis.

[18] Papadopoulos E, Muir C, Russell C, Timmons BW, Falk B, Klentrou P. Markers of biological stress and mucosal immunity during a week leading to competition in adolescent swimmers. J Immunol Res. 2014:234565. http://dx.doi.org/10.1155/2014/234565. 10.1155/2014/234565 PMC4082920 25025080

[19] Gleeson M, Hall ST, McDonald WA, Flanagan AJ, Clancy RL (1999). Salivary IgA subclasses and infection risk in elite swimmers. Immunol Cell Biol.

[20] Li C-Y, Hsu G-S, Suzuki K, Ko M-H, Fang S-H (2015). Salivary Immuno factors, Cortisol and testosterone responses in athletes of a competitive 5,000 m race. Chinese J Physiol.

[21] Cole A, Eastoe J, Cole A, Eastoe J (1988). The oral environment. Biochemistry and oral biology.

[22] Crooks C, Cross M, Wall C, Ali A (2010). Effect of bovine colostrum supplementation on respiratory tract mucosal defenses in swimmers. Int J Sport Nutr Exerc Metab.

[23] Guilhem G, Hanon C, Gendreau N, Bonneau D, Guével A, Chennaoui M (2015). Salivary hormones response to preparation and pre-competitive training of world-class level athletes. Front Physiol.

[24] Inoue H, Ono K, Masuda W, Morimoto Y, Tanaka T, Yokota M, Inenaga K (2006). Gender difference in unstimulated whole saliva flow rate and salivary gland sizes. Arch Oral Biol.

[25] Zouhal H, Jacob C, Delamarche P, Gratas-Delamarche A (2008). Catecholamines and the effects of exercise, training and gender. Sports Med.

[26] Ali N, Pruessner JC (2012). The salivary alpha amylase over cortisol ratio as a marker to assess dysregulations of the stress systems. Physiol Behav.

[27] Boisseau N, Enea C, Diaz V, Dugué B, Corcuff JB, Duclos M (2013). Oral contraception but not menstrual cycle phase is associated with increased free cortisol levels and low hypothalamo-pituitary-adrenal axis reactivity. J Endocrinol Investig.

[28] Kraemer RR, Francois M, Castracane VD (2012). Estrogen mediation of hormone responses to exercise. Metabolism.

[29] Carpenter GH, Proctor GB, Ebersole LE, Garrett JR (2004). Secretion of IgA by rat parotid and submandibular cells in response to autonomimetic stimulation in vitro. Int Immunopharmacol.

[30] Saxon A, Stevens RH, Ramer SJ, Clements PJ, Yu DTY (1978). Glucocorticoids administered in vivo inhibit human suppressor T lymphocyte function and diminish B lymphocyte responsiveness in in vitro immunoglobulin synthesis. J Clin Invest.

